# Pedicle screw malposition following spinal lumbar injury

**DOI:** 10.11604/pamj.2014.17.266.4120

**Published:** 2014-04-12

**Authors:** Ali Akhaddar, El Mehdi Atmane

**Affiliations:** 1 Department of Neurosurgery, Avicenne Military Hospital, Marrakech, Morocco; 2 University of Mohammed V Souissi, Rabat, Morocco; 3 Department of Radiology, Avicenne Military Hospital, Marrakech, Morocco

**Keywords:** Pedicle screw, malposition, spinal lumbar injury

## Image in medicine

Pedicle screws are the main type of instrumentation used in the lumbar spine via the posterior approach. The accuracy of their insertion is always a concern because the malposition of screws is associated with a potential risk of iatrogenic injury of any neurovascular structure they pass. Fortunately, spinal cord and cauda equina injuries are rare. This 18-year-old paraplegic woman presented more than one year after a posterior spinal fusion (extended from L2 to L5 using mono-axial pedicle screws and rods) was performed at another institution following a spinal injury. She complained of atypical mild pain to the lumbar spine, while plain radiographs were suspicious for a L2 left transpedicular screw misplacement. Lumbar spinal CT-scan demonstrated a misplaced L2 left transpedicular screw which penetrated the spinal canal (Figure). The patient and her family were concerned about the intra and perioperative risks of the procedure and denied any further intervention at the moment. Late detection of pedicle screw misplacement into the spinal canal is exceptional and the complexity of revision surgery should be balanced especially if the patient presented with a total paraplegia since the first traumatism. To prevent these serious surgical complications, the surgeon must have a clear understanding of the anatomy of the affected pedicles and to use some imaging guidance (fluoroscopy or radiographs).

**Figure 1:**
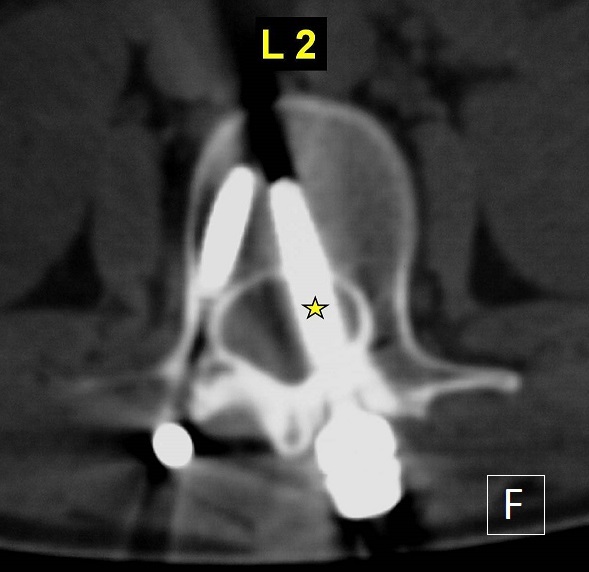
Axial CT-scan (bony window) through the second lumbar vertebrae showing the malpositioned left pedicle screw into the spinal canal (star)

